# Supplementary material for: Bacteria exposed to antiviral drugs develop antibiotic cross-resistance and unique resistance profiles
Source: Commun Biol. 2023 Aug 12;6:837. doi: 10.1038/s42003-023-05177-3 (PMC10423222; doi:10.1038/s42003-023-05177-3)
Supplement: Supplementary file 2 — Supplementary Information [file 42003_2023_5177_MOESM2_ESM.pdf]

Supplementary Figures

Bacteria exposed to antiviral drugs develop antibiotic cross-resistance and unique resistance profiles

Veronica J. Wallace[1], Eric G. Sakowski[1,2], Sarah P. Preheim[1] and Carsten Prasse*[1]

[1]Department of Environmental Health and Engineering, Johns Hopkins University, Baltimore, MD
[2]Department of Science, Mount St. Mary's University, Emmitsburg, MD
*Correspondence: cprasse1@jhu.edu; Tel.: 410-516-5184

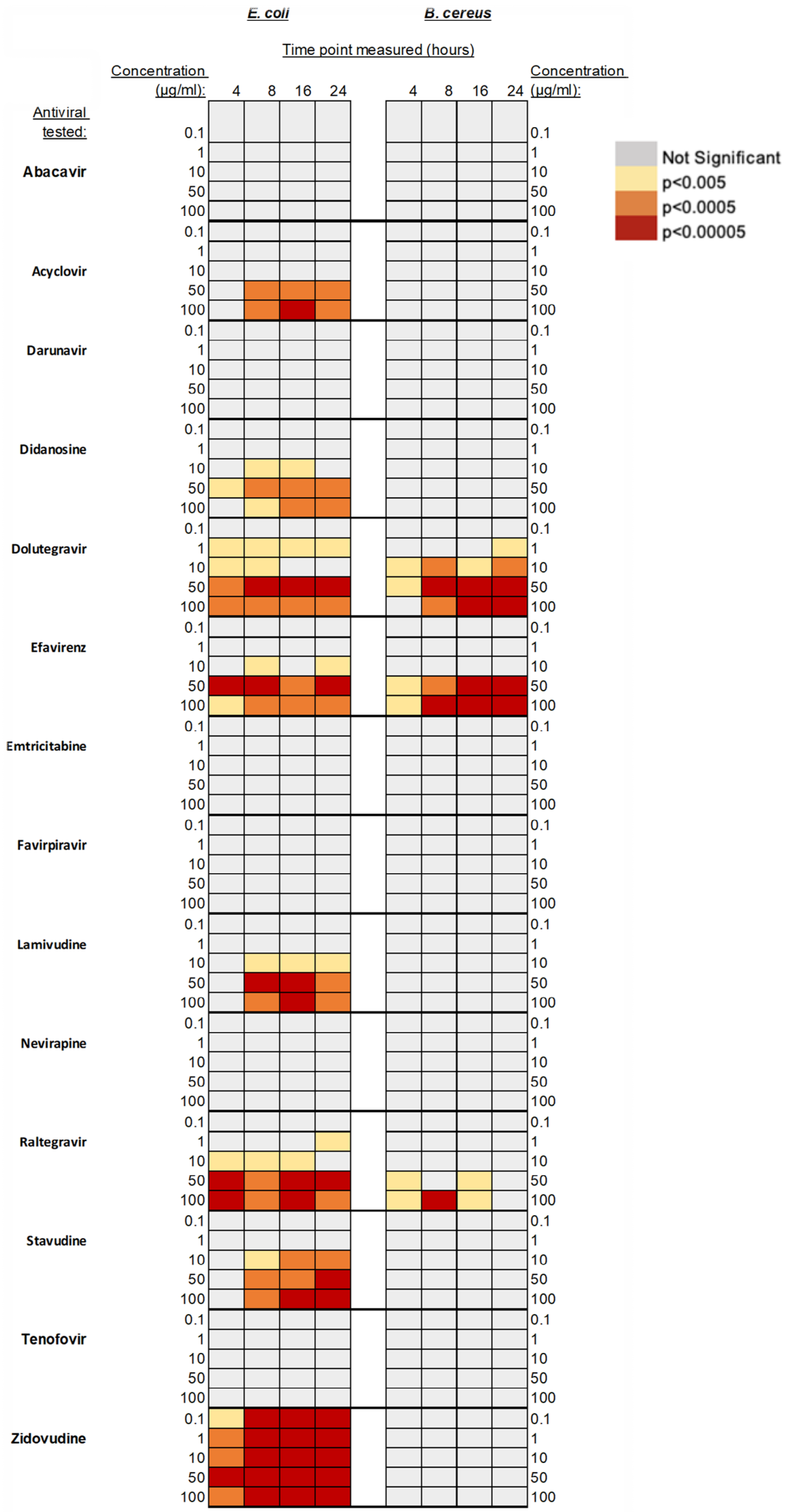

Supplementary Figure 1: Antibacterial effects of all antivirals tested on *E. coli* and *B. cereus*.

(a)

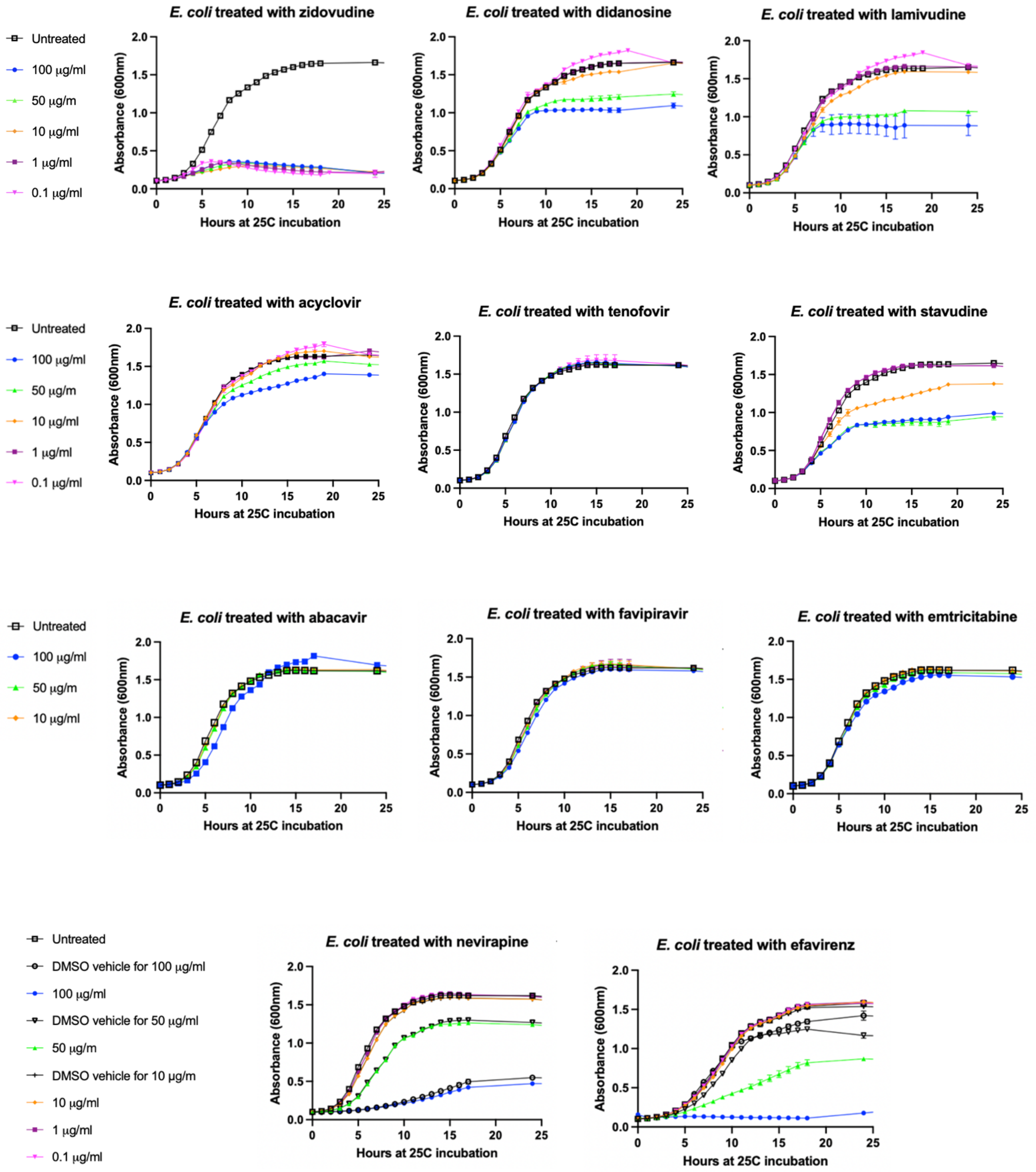
E. coli treated with zidovudine
E. coli treated with didanosine
E. coli treated with lamivudine
E. coli treated with acyclovir
E. coli treated with tenofovir
E. coli treated with stavudine
E. coli treated with abacavir
E. coli treated with favipiravir
E. coli treated with emtricitabine
E. coli treated with nevirapine
E. coli treated with efavirenz
Absorbance (600nm)
Hours at 25C incubation
Untreated
100 μg/ml
50 μg/m
10 μg/ml
1 μg/ml
0.1 μg/ml
DMSO vehicle for 100 μg/ml
DMSO vehicle for 50 μg/ml
DMSO vehicle for 10 μg/m
0.0
0.5
1.0
1.5
2.0
0
5
10
15
20
25

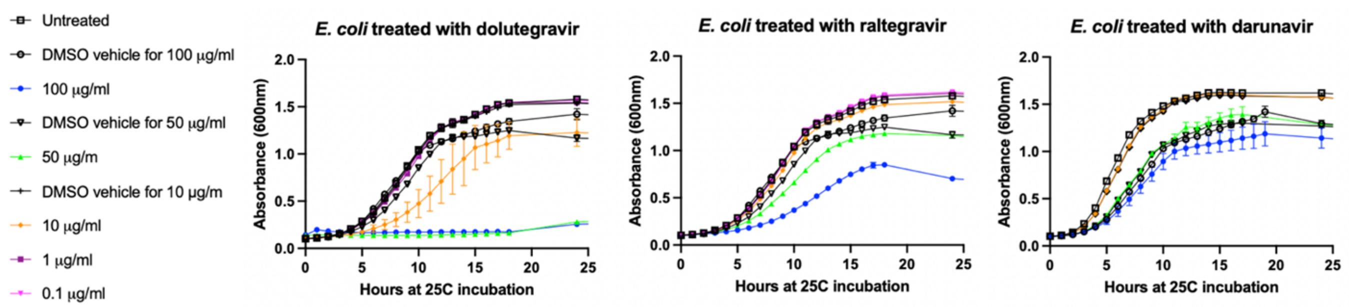
Untreated
DMSO vehicle for 100 µg/ml
100 µg/ml
DMSO vehicle for 50 µg/ml
50 µg/m
DMSO vehicle for 10 µg/m
10 µg/ml
1 µg/ml
0.1 µg/ml
E. coli treated with dolutegravir
E. coli treated with raltegravir
E. coli treated with darunavir
Absorbance (600nm)
Hours at 25C incubation

(b)

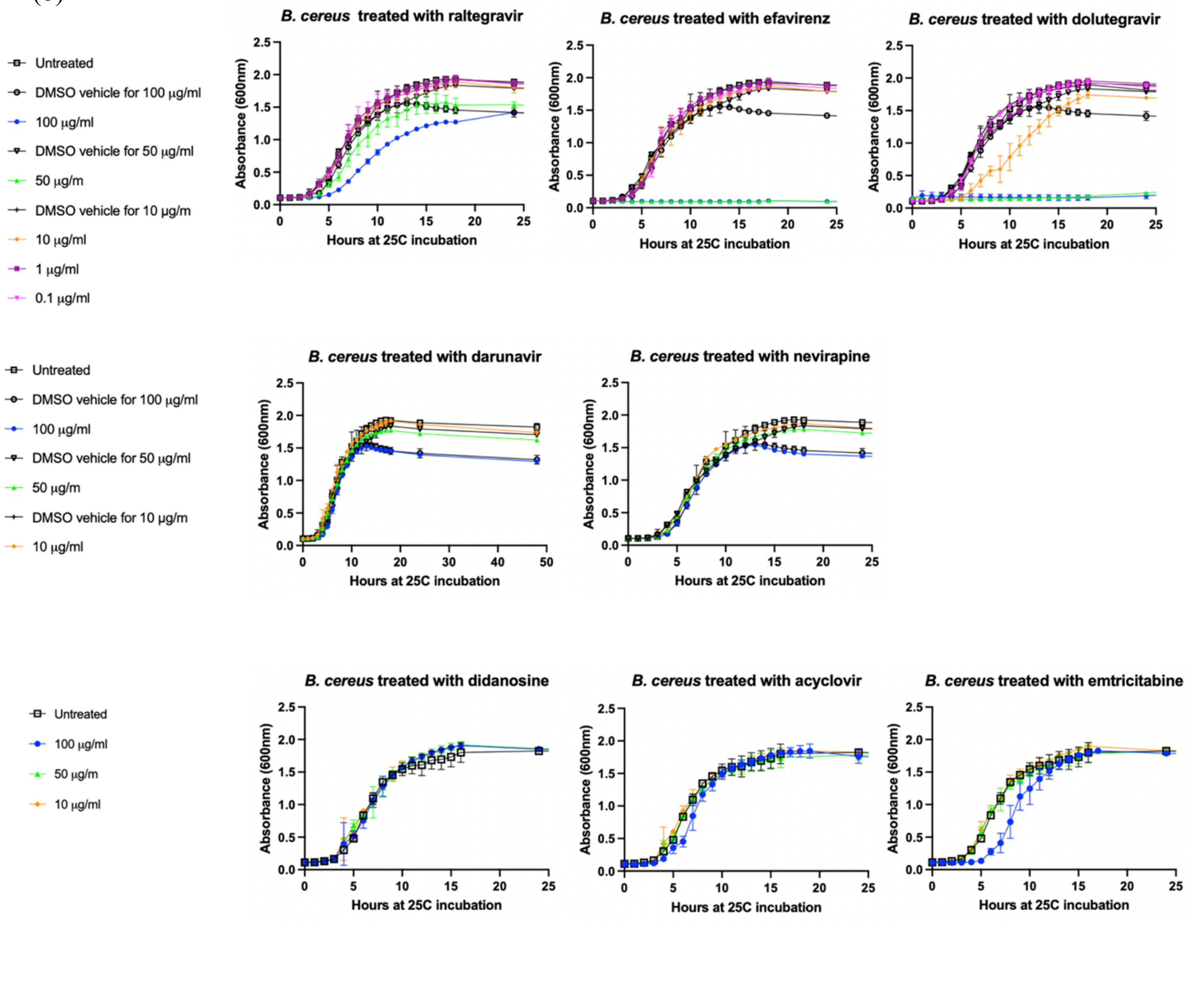
Untreated
DMSO vehicle for 100 µg/ml
100 µg/ml
DMSO vehicle for 50 µg/ml
50 µg/m
DMSO vehicle for 10 µg/m
10 µg/ml
1 µg/ml
0.1 µg/ml
B. cereus treated with raltegravir
B. cereus treated with efavirenz
B. cereus treated with dolutegravir
B. cereus treated with darunavir
B. cereus treated with nevirapine
B. cereus treated with didanosine
B. cereus treated with acyclovir
B. cereus treated with emtricitabine
Absorbance (600nm)
Hours at 25C incubation

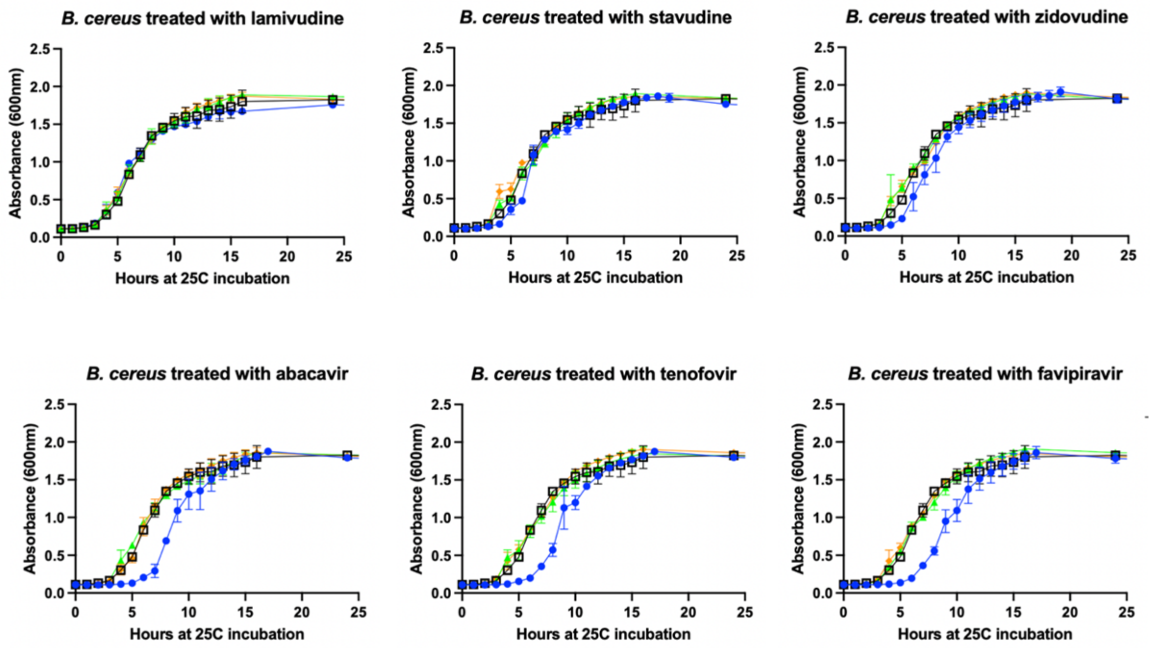

Supplementary Figure 2: Growth of (a) *E. coli* and (b) *B. cereus* in the presence of antivirals, measured as absorbance (600 nm) over 24 hours in 25ºC incubation, as relevant temperature for surface waters and wastewater conditions. Each graph represents data from a separate trial, n=3 technical replicates with the means plotted and error bars demonstrating variation among replicates. Antivirals solubilized in DMSO are compared to DMSO vehicle controls.

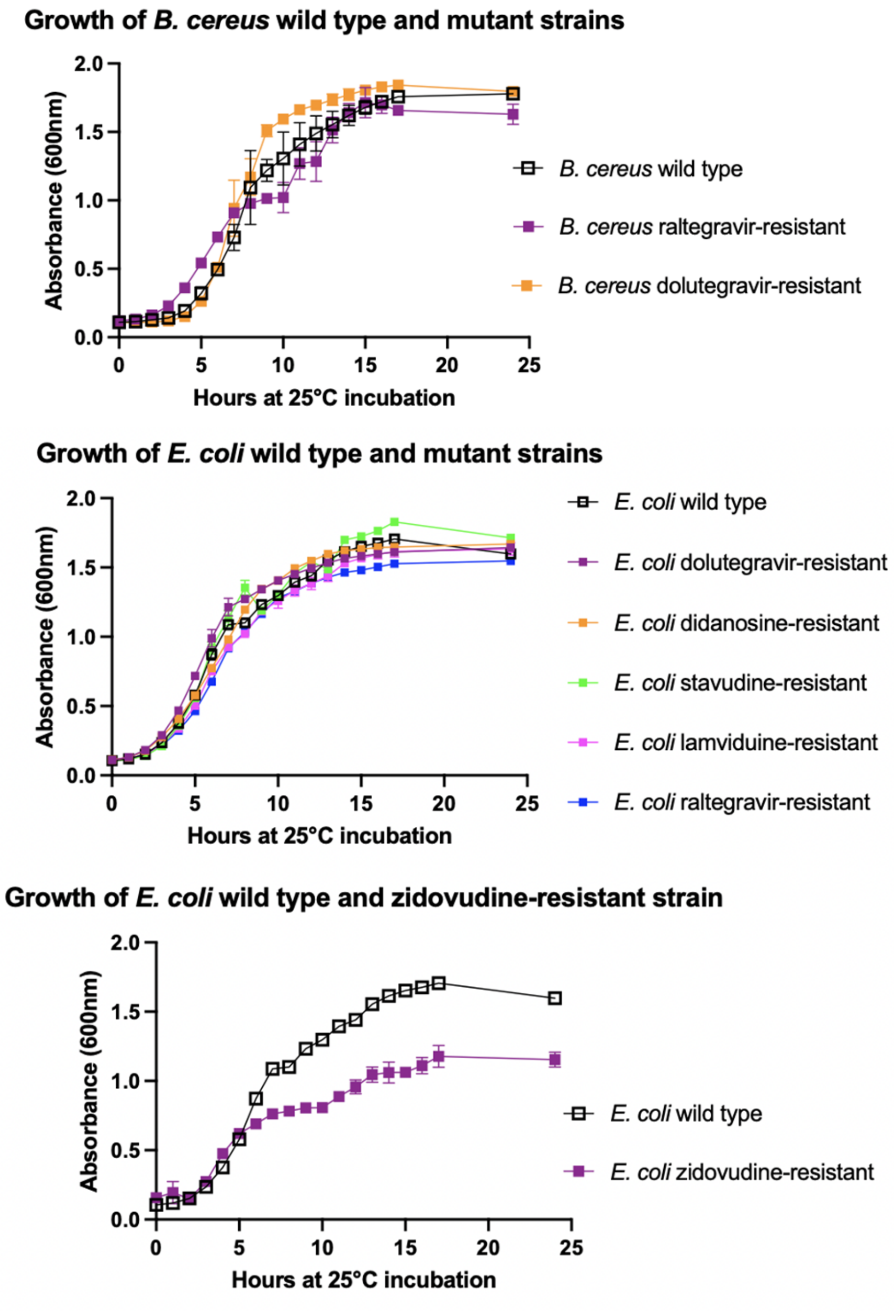

Supplementary Figure 3: Growth of untreated antiviral-resistant mutant strains compared to growth of untreated wild type *E. coli* and *B. cereus*, in the absence of drug treatment.

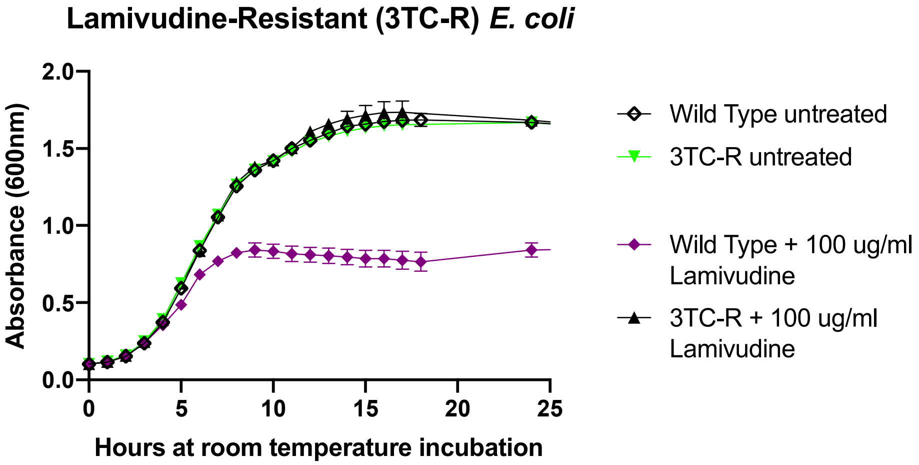

Supplementary Figure 4: Example of the development of antiviral-resistant *E. coli* and *B. cereus*. Antiviral-resistant strains were developed by exposing *E. coli* or *B. cereus* to antivirals for 24 hours. Surviving bacteria were harvested and re-exposed to a higher concentration of the antiviral to test for resistance. For example, *E. coli* treated with 100 µg/ml lamivudine for 24 hours were harvested and re-treated with 100 µg/ml lamivudine. Lamivudine-treated (resistant) *E. coli* show no difference in growth when treated with 100 µg/ml lamivudine compared to untreated, whereas wild type *E. coli* show reduced growth after 5 hours of treatment with 100 µg/ml lamivudine.

(a)

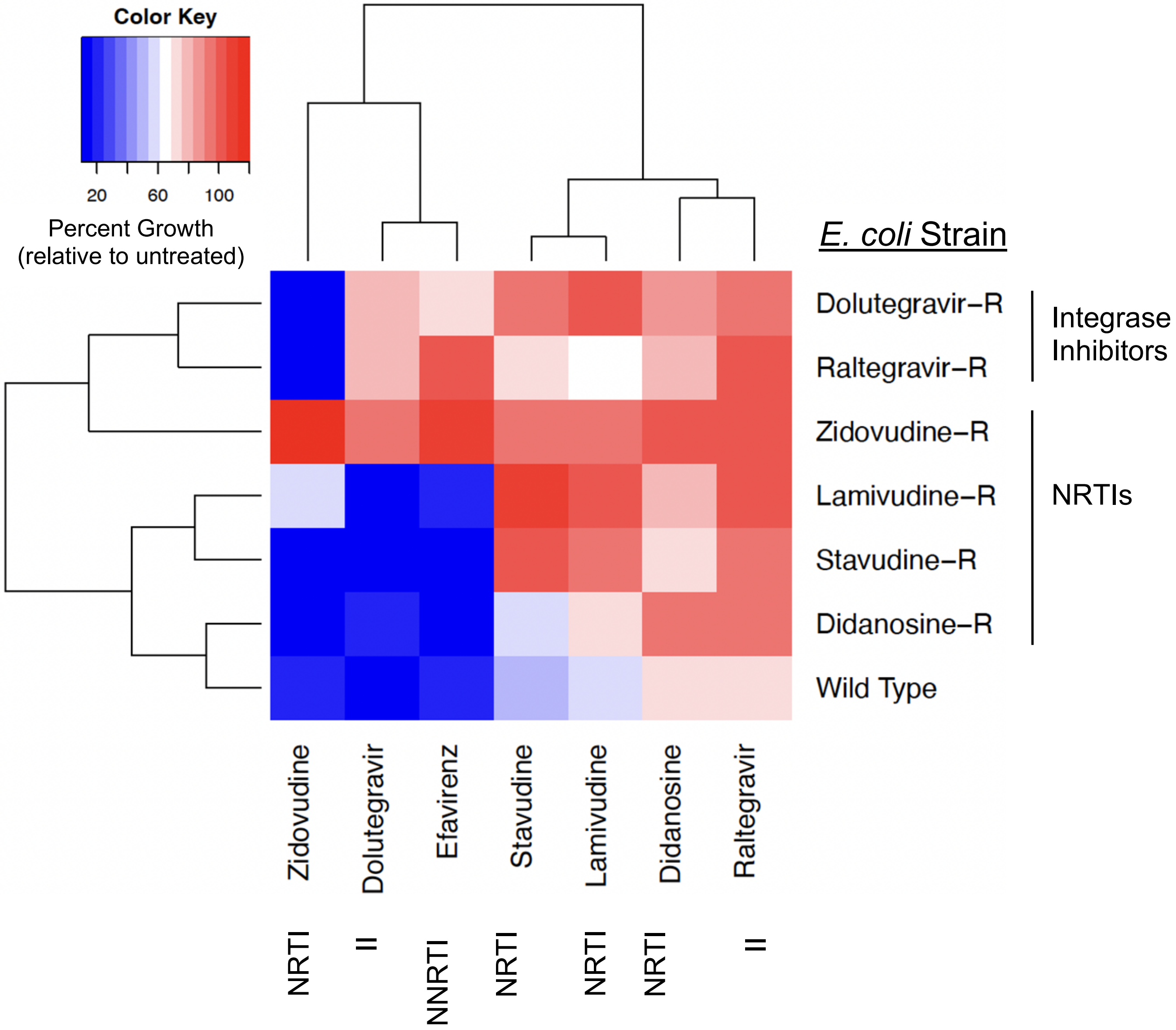
Color Key
20
60
100
Percent Growth
(relative to untreated)
E. coli Strain
Dolutegravir–R
Raltegravir–R
Zidovudine–R
Lamivudine–R
Stavudine–R
Didanosine–R
Wild Type
Integrase Inhibitors
NRTIs
Zidovudine
Dolutegravir
Efavirenz
Stavudine
Lamivudine
Didanosine
Raltegravir
NRTI
II
NNRTI
NRTI
NRTI
NRTI
II

(b)

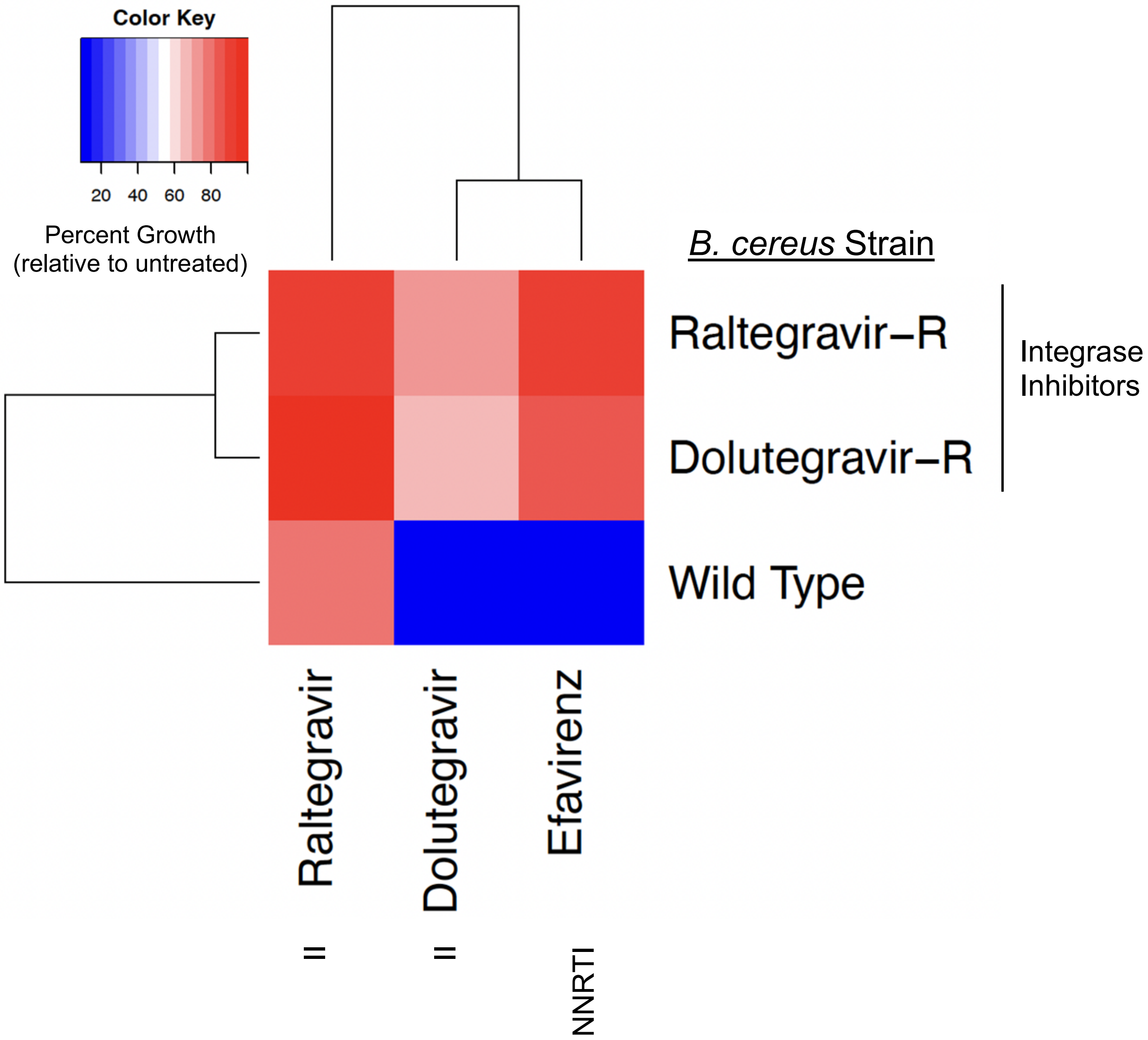

Supplementary Figure 5: Hierarchical clustering (a) for *E. coli* antiviral-resistant strains by antiviral challenge and resistant strain, (b) for *B. cereus* by resistant strain. Antivirals and resistant strains are grouped according to antiviral classification (Nucleoside Reverse Transcriptase Inhibitor (NRTI), Non-Nucleoside Reverse Transcriptase Inhibitor (NNRTI), Integrase Inhibitor (II)).
Mutant and wild-type *E. coli* and *B. cereus* strains were clustered according to their antiviral-resistance profiles. Hierarchical clustering was performed in R (v.3.5.3) using the heatmap.2 function without data scaling in the gplots package (v.3.0.1).
