# Supplementary material for: Bacteria exposed to antiviral drugs develop antibiotic cross-resistance and unique resistance profiles
Source: Commun Biol. 2023 Aug 12;6:837. doi: 10.1038/s42003-023-05177-3 (PMC10423222; doi:10.1038/s42003-023-05177-3)
Supplement: Supplementary file 3 — Description of Additional Supplementary Files [file 42003_2023_5177_MOESM3_ESM.pdf]

Description of Additional Supplementary Files

File name: Supplementary Data 1
Description: Classification, structure and target of antivirals tested.

File name: Supplementary Data 2
Description: Assessing correlations between optical density (OD) measurements from microplate reader and colony forming units (CFU) from agar plates in determination of the impacts of antiviral drugs on the growth of E. coli.

File name: Supplementary Data 3
Description: Results from t-tests to determine significance of antiviral treatments on growth of B. cereus and E. coli over 24 hours.

File name: Supplementary Data 4
Description: Comparing growth rates of untreated wild type vs. antiviral-resistant E. coli and B. cereus.

File name: Supplementary Data 5
Description: Antivirals and concentrations tested for isolation of antiviral-resistant strains, for E. coli and B. cereus.

File name: Supplementary Data 6
Description: Absorbance values at 16-hour timepoint for E. coli or B. cereus antiviral-resistant strains treated with 50 µg/ml antiviral.

File name: Supplementary Data 7
Description: Stability of zidovudine-resistant E. coli and dolutegravir-resistant B. cereus phenotypes over 15 passages.

File name: Supplementary Data 8
Description: Classification and chemical structures of antibiotics tested on antiviral-resistant E. coli and B. cereus.

File name: Supplementary Data 9
Description: Results from t-tests to determine significance of antibiotic effects on antiviral-resistant E. coli and B. cereus compared to effects on wild type E. coli and B. cereus.

File name: Supplementary Data 10
Description: Absorbance values at 16-hour timepoint for E. coli or B. cereus wild type and antiviral-resistant strains treated with antibiotics.

File name: Supplementary Data 11
Description: Absorbance values at specified time points for multidrug-resistant positive control E. coli (ATCC BAA-2471).

File name: Supplementary Data 12
Description: Assembly statistics of wild type and antiviral-resistant E. coli genomes.

File name: Supplementary Data 13
Description: Characteristics of antiviral-resistant E. coli genome mutations.

File name: Supplementary Data 14
Description: Assessment of E. coli BAA-2471 genome for antiviral-resistance mutations.

File name: Supplementary Data 15
Description: Absorbance values (optical density measurements), source data for Figure 5.
